# Systematic Review of Post-Traumatic Parkinsonism, an Emerging Parkinsonian Disorder Among Survivors of Traumatic Brain Injury

**DOI:** 10.1089/neur.2023.0104

**Published:** 2024-01-16

**Authors:** Catherine Rojvirat, Gabriel R. Arismendi, Erin Feinstein, Maynard Guzman, Bruce A. Citron, Vedad Delic

**Affiliations:** ^1^ Laboratory of Molecular Biology, VA New Jersey Health Care System, East Orange, New Jersey, USA.; ^2^ Department of Neurology, VA New Jersey Health Care System, East Orange, New Jersey, USA.; ^3^ Department of Pharmacology, Physiology, and Neuroscience, Rutgers New Jersey Medical School, Newark, New Jersey, USA.; ^4^ Department of Neurology, Rutgers New Jersey Medical School, Newark, New Jersey, USA.

**Keywords:** basal ganglia, loss of consciousness, neurodegeneration, parkinsonism, traumatic brain injury

## Abstract

This systematic review focuses on an increasing subset of traumatic brain injury (TBI) survivors who develop post-traumatic parkinsonism (PTP), characterized by slowness of movement (bradykinesia), rigidity (stiffness), postural instability, and resting tremors caused by obstruction or damage to deep brain structures of the basal ganglia. PTP is rare, and one hypothesis to explain PTP rarity is that TBIs severe enough to affect deep brain structures are often lethal; however, with increasing survivability of TBIs, these numbers are expected to increase. The goal of this review is to raise awareness of an expected global increase of a subgroup of TBI patients who are treatment responsive and report therapeutic results aiding providers in diagnosing, educating, and treating PTP patients. Literature over the past 100 years was considered, and 44,663 peer-reviewed articles were identified. Inclusion criteria required a clinical indication of parkinsonian signs and TBI. Twenty-six case reports were ultimately included from which 36 individual patient data points were extracted for this review. Between 1980 and 2010, there has been an increase in reporting of PTP decade after decade. Forty-seven percent of PTP cases have 1–6 months of latency to symptom onset, and 83% of cases were male. PTP can occur with or without presence of brain lesions, and the most common type of injuries that cause PTP are motor vehicle accidents followed by falls. PTP patients are responsive to surgery or medication treatments. Further detail on PTP symptomology, treatment responsiveness, and injury types is provided.

## Introduction

Traumatic brain injury (TBI) remains the most common neurological disorder and a global health concern.^1^ TBI is defined by the Centers for Disease Control and Prevention as an injury to brain tissue that affects how the brain works resulting from a bump, jolt to the head, or skull penetrating injury. Data from epidemiological studies published between 2015 and 2020 indicate that the global incidence of TBI ranges from 476 per 100,000 in South Korea^2^ to 787 per 100,000 in the United States.^3^ In other words, the incidence of TBI is more than double the combined incidence of stroke, Alzheimer's disease, Parkinson's disease (PD), and multiple sclerosis. Factors affecting TBI survival include pre-hospital care, in-hospital care, access to health insurance, and early intervention.^1^ Although the most drastic TBI survival improvements are reported in low- to middle-income countries, through improved access to healthcare and improved infrastructure, there exists significant room for improvement among wealthy countries with robust healthcare systems. For example, statewide implementation of pre-hospital TBI guidelines in Arizona doubled the survival among patients with severe TBI and tripled survival among patients with TBI requiring intubation.^4^

The new guidelines emphasized improved ventilation and avoidance/treatment of hypotension. Increased TBI survival is followed by years of life with disability. Early and continuous care after a TBI is vital and one of the key takeaways from the 2022 Lancet Neurology Commission on TBI. Access to long-term care and rehabilitation often goes unmet.^5^ The commission has also recommended identifying subgroups of TBI patients most likely to be responsive to specific treatments.^1^ Identifying these groups can improve timely access to effective care and treatments, which will improve recovery and rehabilitation.

Even under the best of circumstances, outcomes after a TBI remain heterogenous.^6^ History of TBI is strongly associated with development of PD years later.^7–11^ PD is typically diagnosed in the sixth decade of life by motor deficits that include progressive slowness of movement (bradykinesia), rigidity (stiffness), postural instability, freezing gait, and resting tremors, collectively referred to as parkinsonism. However, in rare cases, TBI patients can develop post-traumatic parkinsonism (PTP), sharing many of the signs and symptoms of idiopathic PD, after a TBI event. Although TBIs are common, affecting millions of persons each year, the vast majority of TBIs do not produce PTP. The prevailing hypothesis for the rarity of PTP is that TBIs severe enough to compromise the deep brain structures of basal ganglia responsible for orchestrating fine motor movement are often lethal. Motor deficits resulting from brain damage are a common sequelae after a TBI.^12^ Persistent motor weakness resulting specifically from brain lesions after a TBI is also a common finding.^13^ Deficits that cause PTP differ in that they are extrapyramidal in nature, with bradykinesia, rigidity, and resting tremors being the hallmark features. PTP has been described as a disorder of consciousness (DOC) given that loss of consciousness (LOC) often occurs with TBIs and as such may be responsive to amantadine hydrochloride, a prodopaminergic drug commonly used to treat other DOCs.^14^

More recently, the relationship between TBI and parkinsonism has been the subject of reviews that provide insight and offer suggestions for animal modeling to better understand the underpinning biological relationship.^11, 15^ As strategies that improve TBI survival around the world continue to be implemented, the prevalence of PTP is also expected to increase, necessitating an increased awareness and better understanding to expedite treatment for this specific subpopulation of TBI patients.

In most cases, PTP is responsive to pharmacological and surgical intervention, but, if left untreated, may progress to permanent disability. PTP has been reported over the past 100 years, with few published retrospective studies.^16^ Increasing survival of TBI accompanied by neurological deficits, and increased reporting in recent years of PTP, warrants an examination of our current understanding. To this end, we summarized the available PTP literature (over the past 100 years) with a particular focus on the nature of TBIs most likely to cause PTP, varying latency to development of symptoms, sex differences, as well as duration and severity of symptoms. Treatment options for PTP and considerations for future research efforts are presented.

## Methods

**FIG. 1.**
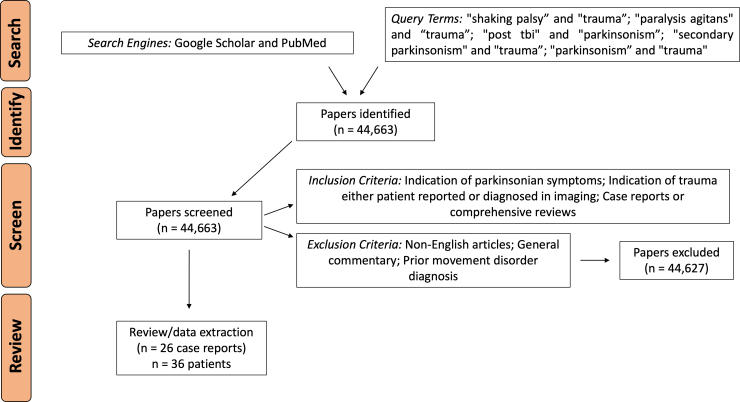
Review strategy and data extraction. Thirty-six cases, from 26 studies, were examined and underwent analysis.

Systematic searches were performed using Google Scholar and PubMed search engines with pre-defined query terms (Fig. 1). A total of 44,663 articles were identified as of February 15, 2023. The final titles were selected based on pre-defined inclusion criteria (indication of parkinsonian signs and indication of trauma, either patient reported or diagnosed in imaging). Twenty-six articles were selected from which 36 cases were extracted, and data were manually extracted from these articles and included in Table 1. Case report data from the 26 studies are presented as percentages of total reports rounded to the nearest whole percent. Prism software (version 9.5.1 for mac OS; GraphPad Software Inc., La Jolla, CA) was used for graphing and generating figures. Lesion cases or lesions are defined as hypointense abnormalities, similar to those observed in stroke or in neurodegenerative disorders. Non-lesion cases or non-lesions are defined as transient subdural hematomas (SDHs) or contusions that compress or transiently obstruct brain regions and resolve after decompression surgeries. A detailed clinical PTP case definition preceding our study was not available. Therefore, definition during literature searches was limited to our inclusion and exclusion criteria because of a dearth of peer-reviewed publications. Case definition as a result of this study is detailed in the Discussion section.

**Table 1.** Reviewed Case Details and Literature Sources

***Authors***	***Year***	***Sex***	***Age***	***Trauma***	***LOC***	***Time of LOC***	***Skull compromised***	***Symptom latency***	***Laterality***	***Classic PD signs***	***Other symptoms***	***Anatomical damage***	***SDH***	***Anatomy categorized***	***Time to treatment***	***Treatment***	***Symptom resolution, latency***
Cobilinschi et al.^26^	2021	M	76	MVA	Y	4 days	Not reported	4 days	Bilateral	Tremor, rigidity		Corticosubcortical midbrain	N	Midbrain	1 day	Levodopa/carbidopa	1 day
Ghoneim et al.^27^	2021	M	55	MVA	NR	Not reported	Not reported	7 years	Not reported	Rigidity	Not reported	Substantia nigra	N	Substantia nigra	Not Reported	Not reported	Not reported
Fukumura^28^	2021	M	52	None	N/A	N/A	N/A	5 weeks	Unilateral	Rigidity, postural instability	Masked facies, stooped posture	Hematoma	Y	Compression of bilateral cerebral cortex, with intact midbrain and basal ganglia	5 weeks	Pramipexole hydrochloride hydrate 0.375 mg per day then subdural drainage catheter	Immediate
Guppy^29^	2018	M	80	Fall	N	N/A	N/A	2 months	Not reported	Postural instability (?), rigidity	Urinary incontinence, stooped posture, shuffling/slow gait, reduced arm swing	Hematoma	Y		2 weeks	Burr hole	3 months
Talh^30^	2017	M	45	Fall	NR	N/A	Not reported	4 weeks	Unilateral	Rigidity, bradykinesia (akenesia?)	Pill rolling	None noted	N	None noted	Not reported	Levodopa/carbidopa	Not reported
Cunningham et al.^31^	2016	F	58	MVA	y	<1 day (NR)	Not reported	4 months	Unilateral to bilateral	Bradykinesia, postural instability, rigidity, tremor	Decreased arm swing, speech problems, masked facies, shuffling/slow gait	Putamen	N	Substantia nigra	Not reported	Levodopa/carbidopa (and other meds) **DBS**	Immediate
Harik^32^	2013	F	23	MVA	Y	1 h	Not reported	3 weeks	Unilateral	Tremor, rigidity, bradykinesia		Substantia nigra	N	Substantia nigra	4 months	Levodopa/carbidopa	Not reported
Gelabert-Gonzalez^33^	2012	M	77	Unclassified TBI	NR	N/A	N/A	2 weeks	Not reported	Rigidity, postural instability, tremor		Hematoma	Y		Not reported	Burr hole	Not reported
Gelabert-Gonzalez^33^	2012	M	71	Unclassified TBI	NR	N/A	N/A	1 week	Not reported	Rigidity, tremor, bradykinesia (akinesia?)		Hematoma	Y		15 days	Levodopa **Burr hole**	7 days
Gelabert-Gonzalez^33^	2012	M	63	None	N/A	N/A	N/A	Unknown	Bilateral	Tremor, bradykinesia	Gait imbalance	Hematoma	Y		Not reported	Burr hole	3 days
Gelabert-Gonzalez^33^	2012	F	59	Unknown	NR	N/A	N/A	Unknown	Bilateral	Bradykinesia, tremor	Speech problems, masked facies	Hematoma	Y		Not reported	Burr hole	4 weeks
Park^34^	2009	M	78	Fall	N	N/A	N/A	3 months	Unilateral	Rigidity, bradykinesia	Speech problems, decreased arm swing	Hematoma	Y		2 weeks	Burr hole	3 weeks
Bostantjopoulou et al.^35^	2009	F	65	Unclassified TBI	NR	N/A	N/A	Unknown	Bilateral	Tremor, rigidity, bradykinesia, postural instability	Headaches, masked facies, speech problems, slow/shuffling gait	Hematoma	Y		Not reported	Burr hole drainage	1 month
Suman^36^	2006	M	81	MVA	NR	N/A	N/A	2 months	Bilateral	Rigidity	Confusion	Hematoma	Y		Not reported	Burr hole	1 week
Kivi et al.^37^	2004	F	59	Pedestrian vs. vehicle	NR	Not reported	Not reported	“Several weeks”	Unilateral	Tremor, rigidity	Speech problems, masked facies	Substantia nigra	N	Substantia nigra	Not reported	Levodopa	Not reported
O'Suilleabhain et al.^38^	2004	M	30s	Pugilism	NR	N/A	Not reported	Unknown	Not reported	Tremor, postural instability		None noted	N	None noted	14 years	Levodopa/carbidopa then, DBS	N/A
Evans et al.^39^	2004	M	27	Fall	Y	Not reported	Not reported	“Months”	Unilateral	Bradykinesia, tremor		Substantia nigra	N	Substantia nigra	Not reported	Levodopa/carbidopa and benzhexol	Not reported
Matsuda et al.^40^	2003	M	51	MVA	Y	Not reported	Not reported	7 months	Unilateral	Rigidity		Midbrain	N	Midbrain	Not reported	Levodopa/carbidopa	6 months
Matsuda et al.^40^	2003	M	27	MVA	Y	Not reported	Not reported	1 year	Unilateral	Rigidity		Midbrain	N	Midbrain	1 year	Levodopa	1 year
Matsuda et al.^40^	2003	M	14	MVA	Y	Not reported	Not reported	3 months	Unilateral	Tremor, rigidity		Midbrain	N	Midbrain	Not reported	Levodopa/benserazide	1 year
Bhatt et al.^41^	2000	M	46	MVA	Y	1 month	Not reported	1 month	Unilateral	Tremor, bradykinesia	Masked facies, increased salivation, speech problems	Putamen, substantia nigra	N	Substantia nigra	Not reported	Levodopa/carbidopa	1 year
Bhatt et al.^41^	2000	M	39	Fall	Y	1 day	Not reported	20 days	Unilateral	Tremor, bradykinesia, rigidity	Masked facies, speech problems, reduced arm swing, slow gait	Substantia nigra	N	Substantia nigra	Not reported	Levodopa/carbidopa	1 year
Bhatt et al.^41^	2000	M	35	MVA	Y	Several hours	Not reported	3 months	Unilateral	Bradykinesia	Micrographia, masked facies	Substantia nigra, subpallidal	N	Substantia nigra	Not reported	Not reported	Not reported
Weist et al.^42^	1999	M	82	Fall	NR	N/A	N/A	Unknown	Unilateral	Not described	Not described	hematoma	Y		1 week	Burr hole	10 days
Weist et al.^42^	1999	M	70	None	N/A	N/A	N/A	Unknown	Unilateral	Bradykinesia, rigidity	Retropulsion, gait instability	Hematoma	Y		2 weeks	Bilateral burr hole	“Weeks”
Weist et al.^42^	1999	M	63	Fall	NR	N/A	N/A	1 week	Not reported	Tremor, rigidity, bradykinesia		Hematoma	Y		3 weeks	Levodopa **Parietal burr hole**	3 months
Doder et al.^43^	1999	M	36	MVA	Y	1 day	Y	6 weeks	Unilateral	Tremor, bradykinesia	Memory impairment	Substantia nigra, putamen, caudate nucleus, internal capsule	N	Substantia nigra	Not reported	Levodopa/carbidopa and anticholinergic	N/A
Sunada^44^	1996	M	75	Unclassified TBI	NR	N/A	N/A	1 month	Bilateral	Tremor, rigidity	Headache, gait disturbance, masked facies, weakness	Hematoma	Y		Not reported	Levodopa **Craniotomy**	3 months
Weiner^45^	1995	M	60s	Pedestrian vs. vehicle	NR	N/A	Not reported	“Months”	Not reported	Not described	Not described	Not reported	N	Not reported	Not reported	Not reported	Not reported
Krul^46^	1987	M	83	Unclassified TBI	N	N/A	N/A	4 weeks	Not reported	Rigidity, tremor	Masked facies, slow/shuffling gait with propulsion and retropulsion	Hematoma	Y		3 weeks	Burr hole	Not reported
Nayernouri^47^	1985	M	37	Fall	Y	10 days	Not reported	3 weeks	Not reported	Bradykinesia, rigidity	Masked facies, shuffling gait	Substantia nigra	N	Substantia nigra	1 month	Levodopa/carbidopa	4 months
Sandyk et al.^48^	1983	F	38	None	N/A	N/A	N/A	Unknown	Bilateral	Tremor, rigidity, bradykinesia	Shuffling/slow gait, headaches	Hematoma	Y		3 weeks	Craniotomy	3 months
Sandyk^49^	1982	M	66	None	N/A	N/A	N/A	4 months	Bilateral	Tremor, bradykinesia,	Gait abnormalities, fatigue, shakiness, masked facies, memory issues, speech problems	Hematoma	Y		4 months	Initially LD, then craniotomy	Immediate
Samiy^50^	1963	M	53	None	N/A	N/A	N/A	4 months	Not reported	Tremor, rigidity	Gait abnormalities without propulsion	Hematoma	Y	Not reported	4 months	Surgical evacuation	3 months
Grimberg^51^	1934	M	45	Pedestrian vs. vehicle	NR	N/A	Not reported	“Immediate”	Not reported	Rigidity, tremor, bradykinesia (?)		Not reported	N	Not reported	Not reported	Not reported	Not reported
Grimberg^51^	1934	M	42	Fall	NR	N/A	Not reported	5 months	Bilateral	Tremor		Not reported	N	Not reported	Not reported	Not reported	Not reported

Data points were extracted from the manuscripts.

DBS, direct brain stimulation; LD, lumbar drain; LOC, loss of consciousness; MVA, motor vehicle accident; N/A, not applicable; NR, not reported; PD, Parkinson's disease; SDH, subdural hematoma; TBI, traumatic brain injury.

### Data sharing

This systematic review article includes all the data points and articles from which the data were extracted in Table 1. Data used to generate graphs are available from the corresponding author upon reasonable request.

## Results

### Post-traumatic parkinsonism reporting increases with increased traumatic brain injury survival

**FIG. 2.**
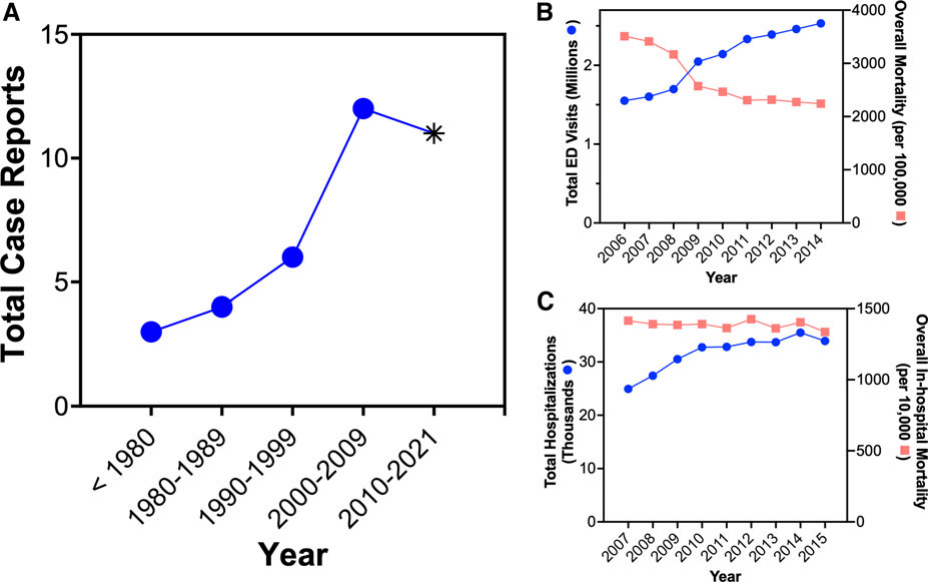
TBI survival and incidence of PTP are increasing. Reporting of PTP in the literature has increased decade after decade from 1980 to 2010. COVID-19 stay-at-home orders issued 23 (**A**). Total emergency room visits for TBI increased from 2006 to 2015,^24^ whereas mortality during the same period for all TBIs has decreased (**B**). Moderate-to-severe TBIs requiring hospitalization increased, whereas in-hospital mortality remained unchanged from 2007 to 2015^25^ (**C**). ED, emergency department; PTP, post-traumatic parkinsonism; TBI, traumatic brain injury.

From 1980 to 2010, the total number of PTP cases has been increasing decade after decade, with a dip in 2020 coinciding with COVID-19 (Fig. 2A). Available published data from 2006 to 2015 report an increase in total TBI emergency room visits and a decrease in total TBI mortality (Fig. 2B). From 2007 to 2015, hospitalizations from moderate-to-severe TBIs increased, whereas mortality remained relatively unchanged during the same period (Fig. 2C).

### Post-traumatic parkinsonism is most commonly caused by injuries from motor vehicle accidents with loss of consciousness and latency to symptom onset of 1–6 months

**FIG. 3.**
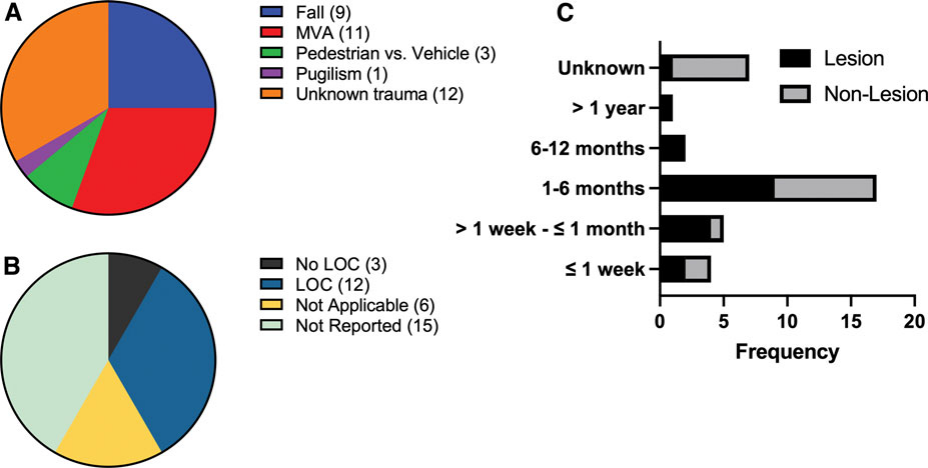
MVAs and falls are the major causes of PTP, with symptom onset most commonly occurring 1–6 months after injury. Unknown TBI (33%), MVA (31%), and falls (25%) are the most common causes of PTP (**A**). Thirty-three percent of PTPs occurred with LOC; 8% of PTPs occurred without LOC (**B**). The most reported latency to symptom onset range is 1–6 months, with 53% of cases reporting the presence of lesions (**C**). ED, emergency department; LOC, loss of consciousness; MVA, motor vehicle accident; PTP, post-traumatic parkinsonism; TBI, traumatic brain injury.

Thirty-one percent of PTP cases occurred because of motor vehicle accidents (MVAs), followed by falls which account for 25% of cases (Fig. 3A). Many PTP cases were of unknown trauma cause, although trauma was indicated in 33% (Fig. 3A). LOC was reported in 33% of cases, whereas only 8% of PTP cases reported no LOC (Fig. 3B). Forty-two percent of PTP cases did not report LOC (Fig. 3B). PTP symptom onset is most commonly reported 1–6 months after injury, with 53% of cases occurring with the presence of lesions (Fig. 3C).

### Post-traumatic parkinsonism can occur in all age groups, with and without the presence of lesions, and most frequently in male traumatic brain injury patients

**FIG. 4.**
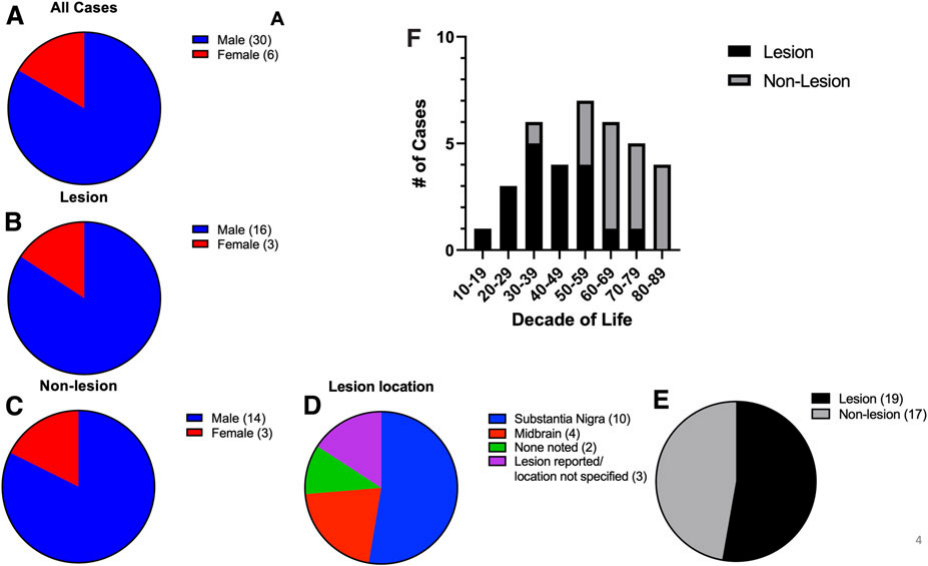
PTP occurs most frequently in males between the ages of 50 and 59, and the diagnosis is accompanied with a report of lesion in the substantia nigra pars compacta. PTP with or without identified brain lesions is reported 5 times more in males than females (**A–C**). Fifty-three percent of lesions in PTP are found in the substantia nigra (**D**). Fifty-three ppercent of all PTP cases have an identified brain lesion (**E**). The proportion of non-lesion to lesion PTP cases increases over the age of 50 (**F**). PTP, post-traumatic parkinsonism.

Fifty-three percent of PTP cases had a confirmed lesion (Fig. 4E). Fifty-three percent of lesions were localized specifically to the substantia nigra whereas 21% were reported more broadly in the midbrain (Fig. 4D). PTP with lesions occur more frequently in patients under the age of 60 (Fig. 4F). Males are 5 times more likely to develop PTP, regardless of lesion presence or absence (Fig. 4A–C).

### Post-traumatic parkinsonism can have parkinsonian symptomology with good treatment responsiveness

**FIG. 5.**
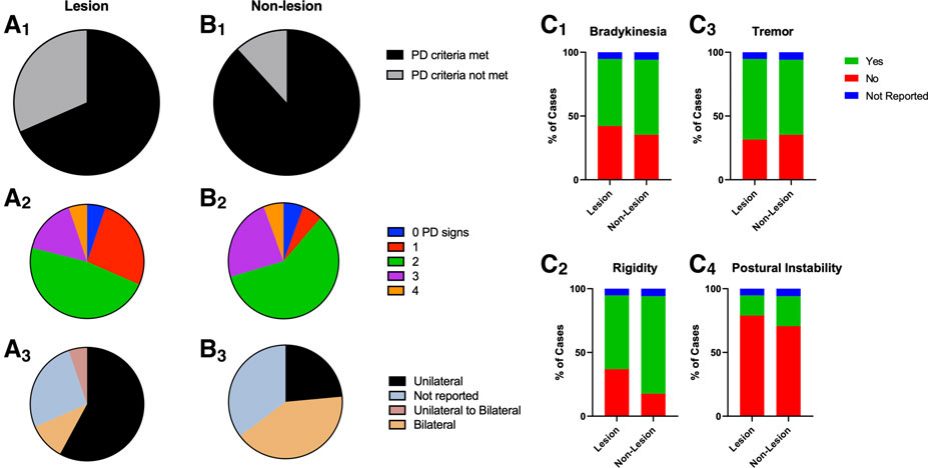
Majority of PTP cases met at least two parkinsonian signs. Sixty-eight percent of PTP cases with an identified lesion met PD diagnostic criteria of at least two parkinsonian signs (**A1**). Forty-seven percent of cases had two parkinsonian signs, 16% had three and 5% had four signs (**A2**). Fifty-eight percent of PTP cases with lesion(s) had unilateral signs, 11% had bilateral signs, 5% had unilateral to bilateral signs, and in 26% of cases laterality was not reported (**A3**). Eighty-eight percent of PTP cases with no identified lesion met PD diagnostic criteria of at least two parkinsonian signs (**B1**). Fifty-nine percent of non-lesion PTP had two signs, 24% had three signs, and 6% had four signs (B2). Twenty-four percent of non-lesion PTP cases had unilateral signs, 41% had bilateral signs, and in 35% of PTP cases symptom and sign laterality were not reported (**B3**). Bradykinesia was present in 52% of PTP cases with lesion(s) and 59% of non-lesion cases (**C1**). Rigidity was reported in 58% of lesion PTP cases and 76% of non-lesion PTP cases (**C2**). Tremors were reported in 63% of lesion and 58% of non-lesion PTP cases (**C3**). Postural instability was reported in 16% of lesion and in 24% of non-lesion cases (**C4**). PD, Parkinson's disease; PTP, post-traumatic parkinsonism.

**FIG. 6.**
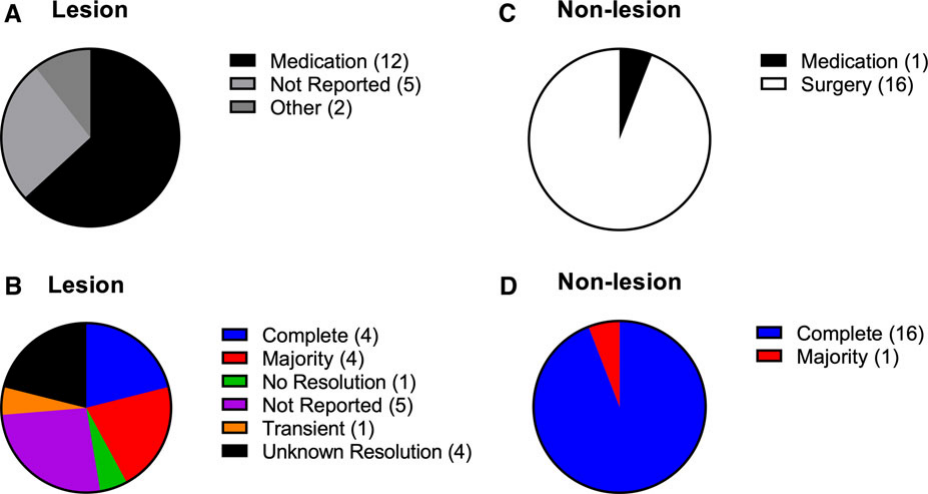
PTP patients are responsive to surgery or medication treatments. Carbidopa/levodopa was the predominant treatment for PTP patients with identified lesion(s) (**A**). More than 47% of patients reported complete, majority, or transient recovery in response to medication (**B**). For patients with non-lesion PTP, surgery (decompression) was the predominant treatment choice (**C**). Ninety-four percent of non-lesion PTP cases had complete resolution after surgical intervention (**D**). PTP, post-traumatic parkinsonism.

Sixty-eight percent of lesion PTP (Fig. 5A_1_–A_2_) and 88% of non-lesion PTP (Fig. 5B_1_–B_2_) cases met at least two of the four common parkinsonian diagnostic criteria (bradykinesia, rigidity, tremor, and postural instability). Listed in descending order of sign prevalence for lesion PTP are: tremors 63% (Fig. 5C_3_); rigidity 58% (Fig. 5C_2_); bradykinesia 52% (Fig. 5C_1_); and postural instability 16% (Fig. 5C_4_). Prevalence of signs for non-lesion PTP in descending order are: rigidity (Fig. 5C_2_); bradykinesia 59% (Fig. 5C_1_); tremors 58% (Fig. 5C_3_); and postural instability 24% (Fig. 5C_4_). Lesion PTP had mostly unilateral presentation (Fig. 5A_3_) whereas non-lesion PTP had mostly bilateral presentation (Fig. 5B_3_). PTP with lesions were responsive to medication treatment (carbidopa/levodopa), with >47% of cases having reported complete, majority, or transient recovery (Fig. 6A,B). Patients with non-lesion PTP were responsive to surgical intervention to alleviate compression caused by SDHs (Fig. 6C). Ninety-four percent of non-lesion PTP patients reported complete resolution of symptoms after decompression surgery (Fig. 6D).

## Discussion

Impetus for this systematic review comes from an increase in TBI survival and our ongoing studies into the biological relationship between the history of TBI and PD. Investigation of TBI literature indicated that, along with an increase in TBI survival, there is an increase in reports of PTP. Both the number of TBI survivors and persons with consequences after a TBI are expected to increase, and according to the 2022 Lancet Neurology Commission on TBI, identifying TBI subgroups responsive to specific treatment is an effective strategy to address this growing problem.^1^ Patients who develop PTP are one such group, sharing presentation and treatment options with parkinsonian disorders. As such, it is important to better understand this group to provide more effective treatments and help design more effective research studies by identifying knowledge gaps that remain.

### Increase in post-traumatic parkinsonism reporting coincides with increase in traumatic brain injury survival

The prevailing hypothesis for the rarity of PTP is that injuries severe enough to cause PTP are lethal. It stands to reason that if TBI survival is improved that there would also be an increase in PTP reporting. Indeed, we observe that between 1980 and 2009 there has been a steep increase in PTP reporting (Fig. 2A) coinciding with improvements in healthcare that lead to an increase in TBI survival (Fig. 2B,C). If this trend continues, we expect to observe a continued increase in PTP reporting. The 2022 Lancet Neurology Commission on TBI recommended that identifying TBI subgroups responsive to specific treatment is an effective strategy to help address the growing number of TBI survivors with lifelong disabilities.^1^

### Improved case definition

Based on the data collected from case reports, a clearer clinical definition of PTP is emerging, as a parkinsonian disorder that most frequently occurs in men (83%), 1–6 months after a TBI event, and having at least two of the four common parkinsonian signs. Bradykinesia, rigidity, and tremors are common in PTP with or without identified lesion(s), whereas postural instability is reported only in 16% of lesion and 24% of non-lesion PTP cases. Though PTP can occur at any age between 10 and 89, most cases were over the age of 29 (Fig. 4F). MVAs were the most common cause of reported PTP cases followed by falls (Fig. 3A). Without definitive biomarkers, a clinical TBI history remains paramount to distinguish PTP from other parkinsonian disorders, particularly among persons over the age of 50, when other parkinsonian disorders are likely to develop. Neuroimaging findings show either an SDH causing transient compressive obstruction of the basal ganglia or hypointense lesions confined to the basal ganglia, similar to what is observed in neurodegenerative diseases. These are referred to as lesion versus non-lesion PTP. SDH PTP is resolved by decompressive surgery, and lesion PTP is responsive to dopamine replacement therapy (DRT).

Some missing details could be filled with more complete patient history. For instance, in 33% of published PTP cases, the specific trauma type was unknown (Fig. 3A). Similarly, state of consciousness (SOC) was not reported in 42% of cases (Fig. 3B). Inclusion of trauma causes and SOC histories in future reporting would significantly improve our understanding of PTP and help us further refine a clinical definition.

### Post-traumatic parkinsonism treatments responsiveness and outlook

PTP is responsive to DRT in cases where lesions were detected (Fig. 6A,B), whereas decompression surgeries were effective in cases where hematomas were present (Fig. 6C,D). Forty-two percent of lesion cases had complete or majority resolution of symptoms (Fig. 6B) whereas lesion cases that required decompression surgery had 94% complete and 6% had majority symptom resolution (Fig. 6D). We delineate between PTP cases that have detectable lesions on neuroimaging and those with transient hematomas as structural abnormalities, which we refer to as non-lesion. Lesions were not reported in cases where hematomas were detected and managed, resulting in resolution or improvement of symptoms.

It is important to recognize that in order to develop future targeted therapeutics for PTP, better reporting of long-term PTP outcomes from current treatments (e.g., surgery and DRT) needs to be implemented. To emphasize, surgical intervention had better, 100% reporting overall of complete or major symptom resolution (Fig. 6C,D); by comparison, 53% of lesion cases reported complete, major, transient, or no resolution (Fig. 6B). None of the published cases report long-term follow-up to determine long-term patient outcomes. A fundamental question remains unknown, whether parkinsonism in treatment-responsive patients resolves or worsens with time. Moreover, it remains unknown whether patients placed on medication(s) remain on symptomatic treatment indefinitely.

### Future research direction

It remains unknown whether patients responsive to treatment continued the treatment indefinitely or whether clinical benefits persisted after treatment cessation. In other words, it is unknown whether post-PTP is progressive or static. Additionally, it remains unclear whether PTP occurred during the prodromal period of PD pathology already present in the patient. TBIs, resulting mostly from falls, increase in patients before their PD diagnosis, likely attributable to undiagnosed motor deficits.^17^ Future studies with the aid of effective biomarkers will be required to better understand this dynamic. Because of the clear link to compromised basal ganglia, PTP patients are of particular interest to determine whether they are at a higher risk for developing PD later in life. PTP is delayed from the point of injury by up to a year, and maybe even longer, which impedes diagnosis and treatment. PTP is responsive to PD treatments and does not follow rapid progression like other more aggressive parkinsonian disorders. In the absence of definitive diagnostic biomarkers in living patients, many cases of PD linked to TBI may be misdiagnosed as PD instead of PTP. Identifying additional cases of PTP could present an opportunity to study the effectiveness of drugs to slow or stop PTP progression or development of PD after PTP. Currently, these drugs are tested in persons who have developed PD. Alpha synuclein (α-Syn) amplification assay technology has been shown to detect PD biomarkers in the cerebrospinal fluid of PD patients.^18,19^

More recently, these assays have been used to differentiate between leucine-rich repeat kinase-2 variant and idiopathic PD.^20^ α-Syn amplification assays have the potential to distinguishing between PTP and idiopathic PD, particularly in patients >60.

One theme confirmed in this review involves the diversity of responses to TBI. Genetic variants could account for many differences in post-injury neurodegeneration. Many genes have already been connected directly to parkinsonian disorders. Variants within these and other genes could help predict potential post-injury outcomes^21^ and identify therapeutic targets to explore. It remains unclear whether pre-clinical models accurately predict the mechanism of PD progression after PTP. However, if true, those developed therapeutics may prove useful for patients long term and therefore is an important area of continued research to prevent future disability in PTP patients. We have found in pre-clinical rat models of α-Syn fibril-induced PD that pTau is localized in Lewy bodies (LBs) within dopaminergic neurons.^22^ Similarly, we also found pTau aggregates within dopaminergic neurons of rats that underwent repetitive mild TBI.^22^ If PTP induces pTau aggregation in surviving dopaminergic neurons, it could predispose cells to development of PD-specific proteinopathy by cross-seeding aggregation of α-Syn into LBs and Lewy neurites. Similarly, pTau-targeted therapeutics developed for Alzheimer's disease could potentially be used to limit or slow the progression of PTP to PD.

### Limitations

Although PTP case reports have increased decade after decade, the number reported in the literature is still small, totaling 36 reported cases from 26 studies. Some cases of PTP that develop after 1 year, particularly in patients >60, may be misdiagnosed as PD, thereby limiting the numbers of PTP cases. Long-term follow-up of PTP cases beyond 1 year can further delineate between PTP and PD in older persons, but is absent from the literature. The majority of PTP case reports did not specify the cause of trauma.

## Conclusion

Increase in PTP reporting decade after decade is likely a result of increased TBI incidence and increased TBI survival. These numbers are expected to rise. PTP is responsive to either DRTs or surgeries depending on whether PTP is a result of a lesion or from an SDH. Males are 5 times more likely to develop PTP, and the most common injury type that causes PTP is an MVA with LOC. PTP occurs most commonly after a 1- to 6-month latency period. More comprehensive reporting and long-term follow-up studies are needed to determine whether PTP is static or progressive.

## Transparency, Rigor, and Reproducibility Summary

This systematic review was designed to summarize clinical reports of post-traumatic parkinsonism that occurs after a traumatic brain injury. Systematic searches were performed using Google Scholar and PubMed search engines with pre-defined query terms (Fig. 1). A total of 44,663 manuscripts were identified as of February 15, 2023. The final titles were selected based on pre-defined inclusion criteria (indication of parkinsonian signs and symptoms, and indication of trauma either patient reported or diagnosed in imaging). Twenty-six manuscripts were selected from which 36 cases were extracted, and data were manually extracted from these manuscripts and included in Table 1. Case report data from the 26 studies are presented as percentages of total reports rounded to the nearest whole percent. Prism version 9.5.1 for mac OS was used for graphing and generating figures. Inclusion criteria were not pre-registered. Any ambiguity about article inclusion was discussed among authors, and agreement was reached before inclusion.

## Abbreviations Used

α-Syn: alpha-synuclein
DOS: disorder of consciousness
DRT: dopamine replacement therapy
LBs: Lewy bodies
LOC: loss of consciousness
MVA: motor vehicle accident
PD: Parkinson's disease
PTP: post-traumatic parkinsonism
SDH: subdural hematoma
SOC: state of consciousness
TBI: traumatic brain injury

## References

[1] Maas AIR, Menon DK, Manley GT, et al.; InTBIR Participants and Investigators. Traumatic brain injury: progress and challenges in prevention, clinical care, and research. Lancet Neurol 2022;21(11):1004–1060; doi: 10.1016/S1474-4422(22)00309-X 36183712 PMC10427240

[2] Kim HK, Leigh JH, Lee YS, et al. Decreasing incidence and mortality in traumatic brain injury in Korea, 2008–2017: a population-based longitudinal study. Int J Environ Res Public Health 2020;17(17):6197; doi: 10.3390/ijerph17176197 32859061 PMC7504501

[3] Taylor CA, Bell JM, Breiding MJ, et al. Traumatic brain injury-related emergency department visits, hospitalizations, and deaths—United States, 2007 and 2013. MMWR Surveill Summ 2017;66(9):1–16; doi: 10.15585/mmwr.ss6609a1 PMC5829835 28301451

[4] Spaite DW, Bobrow BJ, Keim SM, et al. Association of statewide implementation of the prehospital traumatic brain injury treatment guidelines with patient survival following traumatic brain injury: the Excellence in Prehospital Injury Care (EPIC) Study. JAMA Surg 2019;154(7):e191152; doi: 10.1001/jamasurg.2019.1152 31066879 PMC6506902

[5] Andelic N, Roe C, Tenovuo O, et al. Unmet rehabilitation needs after traumatic brain injury across Europe: results from the CENTER-TBI Study. J Clin Med. 2021;10(5):1035; doi: 10.3390/jcm10051035 33802336 PMC7959119

[6] Katz DI, Bernick C, Dodick DW, et al. National Institute of Neurological Disorders and Stroke consensus diagnostic criteria for traumatic encephalopathy syndrome. Neurology 2021;96(18):848–863; doi: 10.1212/WNL.0000000000011850 33722990 PMC8166432

[7] Crane PK, Gibbons LE, Dams-O'Connor K, et al. Association of traumatic brain injury with late-life neurodegenerative conditions and neuropathologic findings. JAMA Neurol 2016;73(9):1062–1069; doi: 10.1001/jamaneurol.2016.1948 27400367 PMC5319642

[8] Kukull WA, Higdon R, Bowen JD, et al. Dementia and Alzheimer disease incidence: a prospective cohort study. Arch Neurol 2002;59(11):1737–1746; doi: 10.1001/archneur.59.11.1737 12433261

[9] Bennett DA, Schneider JA, Arvanitakis Z, et al. Overview and findings from the religious orders study. Curr Alzheimer Res 2012;9(6):628–645; doi: 10.2174/156720512801322573 22471860 PMC3409291

[10] Bennett DA, Schneider JA, Buchman AS, et al. Overview and findings from the rush Memory and Aging Project. Curr Alzheimer Res 2012;9(6):646–663; doi: 10.2174/156720512801322663 22471867 PMC3439198

[11] Delic V, Beck KD, Pang KCH, et al. Biological links between traumatic brain injury and Parkinson's disease. Acta Neuropathol Commun 2020;8(1):45; doi: 10.1186/s40478-020-00924-7 32264976 PMC7137235

[12] Walker WC, Pickett TC. Motor impairment after severe traumatic brain injury: a longitudinal multicenter study. J Rehabil Res Dev 2007;44(7):975–982; doi: 10.1682/jrrd.2006.12.0158 18075954

[13] Choi GS, Kim OL, Kim SH, et al. Classification of cause of motor weakness in traumatic brain injury using diffusion tensor imaging. Arch Neurol 2012;69(3):363–367; doi: 10.1001/archneurol.2011.1930 22083801

[14] Formisano R, Zasler ND. Posttraumatic parkinsonism. J Head Trauma Rehabil 2014;29(4):387–390; doi: 10.1097/HTR.0000000000000027 24695262

[15] Padmakumar S, Kulkarni P, Ferris CF, et al. Traumatic brain injury and the development of parkinsonism: understanding pathophysiology, animal models, and therapeutic targets. Biomed Pharmacother 2022;149:112812; doi: 10.1016/j.biopha.2022.112812 35290887 PMC9050934

[16] Krauss JK, Trankle R, Kopp KH. Post-traumatic movement disorders in survivors of severe head injury. Neurology 1996;47(6):1488–1492; doi: 10.1212/wnl.47.6.1488 8960732

[17] Camacho-Soto A, Warden MN, Searles Nielsen S, et al. Traumatic brain injury in the prodromal period of Parkinson's disease: a large epidemiological study using medicare data. Ann Neurol 2017;82(5):744–754; doi: 10.1002/ana.25074 29024046 PMC5812286

[18] Russo MJ, Orru CD, Concha-Marambio L, et al. High diagnostic performance of independent alpha-synuclein seed amplification assays for detection of early Parkinson's disease. Acta Neuropathol Commun 2021;9(1):179; doi: 10.1186/s40478-021-01282-8 34742348 PMC8572469

[19] Russo MJ, Orru CD, Concha-Marambio L, et al. Correction to: High diagnostic performance of independent alpha-synuclein seed amplification assays for detection of early Parkinson's disease. Acta Neuropathol Commun 2021;9(1):190; doi: 10.1186/s40478-021-01292-6 34836545 PMC8620217

[20] Siderowf A, Concha-Marambio L, Lafontant DE, Farris CM, Ma Y, Urenia PA, et al.; Parkinson's Progression Markers Initiative. Assessment of heterogeneity among participants in the Parkinson's Progression Markers Initiative cohort using alpha-synuclein seed amplification: a cross-sectional study. Lancet Neurol 2023;22(5):407–417; doi: 10.1016/S1474-4422(23)00109-6 37059509 PMC10627170

[21] McDevitt J, Krynetskiy E. Genetic findings in sport-related concussions: potential for individualized medicine? Concussion 2017;2(1):CNC26; doi: 10.2217/cnc-2016-0020 30202567 PMC6096436

[22] Delic V, Karp JH, Guzman M, et al. Repetitive mild TBI causes pTau aggregation in nigra without altering preexisting fibril induced Parkinson's-like pathology burden. Acta Neuropathol Commun 2022;10(1):170; doi: 10.1186/s40478-022-01475-9 36435806 PMC9701434

[23] Moreland A, Herlihy C, Tynan MA, et al.; CDC Public Health Law Program; CDC COVID-19 Response Team, Mitigation Policy Analysis Unit. Timing of state and territorial COVID-19 stay-at-home orders and changes in population movement—United States, March 1–May 31, 2020. MMWR Morb Mortal Wkly Rep 2020;69(35):1198–1203; doi: 10.15585/mmwr.mm6935a2 32881851 PMC7470456

[24] Peterson AB, Xu L, Daugherty J, et al.; Centers for Disease Control and Prevention. Surveillance report of traumatic brain injury-related emergency department visits, hospitalizations, and deaths—United States, 2014. Centers for Disease Control and Prevention, U.S. Department of Health and Human Services; 2019.

[25] Alinani A, Mills B, Gause E, et al. National Institutes of Health clinical research funding and all-cause in-hospital traumatic brain injury-related mortality. Cureus 2022;14(7):e27228; doi: 10.7759/cureus.27228 36035060 PMC9400552

[26] Cobilinschi C, Popa A, Scărlătescu F, et al. Sudden onset of Parkinson's disease after traumatic brain injury—an unusual cause of difficult weaning. Rom J Emerg Surg 2021;3(1):3–10.

[27] Ghoneim A, Pollard C, Tyagi A, et al. Substantia nigra micro-haemorrhage causing ipsilateral unilateral Parkinsonism and abnormal dopamine transporter scan uptake. BJR Case Rep 2021;7(1):20200118; doi: 10.1259/bjrcr.20200118 33614119 PMC7869125

[28] Fukumura M, Murase S, Kuroda Y, et al. Secondary parkinsonism caused by chronic subdural hematomas owing to compressed cortex and a disturbed cortico-basal ganglia-thalamocortical circuit: illustrative case. J Neurosurg Case Lessons 2021;1(24):CASE21216; doi: 10.3171/CASE21216 35855096 PMC9245839

[29] Guppy KH, Khandhar SM, Ochi C. Reversible parkinson-like symptoms in patient with bilateral chronic subdural hematomas and cervical spinal stenosis. World Neurosurg 2018;109:285–290; doi: 10.1016/j.wneu.2017.10.009 29038083

[30] Abu Talh K, Sulaiman M, Joshi D, et al. Development of Parkinsonism symptoms immediately after severe head injury. Neurosciences (Riyadh) 2017;22(4):308–310; doi: 10.17712/nsj.2017.4.20170240 29057858 PMC5946382

[31] Cunningham MG, Yadollahikhales G, Vitaliano G, et al. Administration of electroconvulsive therapy for depression associated with deep brain stimulation in a patient with post-traumatic Parkinson's Disease: a case study. BMC Psychiatry 2016;16(1):399; doi: 10.1186/s12888-016-1108-y 27842519 PMC5109836

[32] Harik SI, Al-Hinti JT, Archer RL, et al. Hemiparkinsonism after unilateral traumatic midbrain hemorrhage in a young woman. Neurol Clin Pract 2013;3(1):4–7; doi: 10.1212/CPJ.0b013e318283fef6 29406522 PMC5765937

[33] Gelabert-Gonzalez M, Serramito-García R, Aran-Echabe E. Parkinsonism secondary to subdural haematoma. Neurosurg Rev 2012;35(3):457–460; discussion, 460–461; doi: 10.1007/s10143-012-0386-1 22527627

[34] Park B, Song SK, Hong JY, et al. Parkinsonsim due to a chronic subdural hematoma. J Mov Disord 2009;2(1):43–44; doi: 10.14802/jmd.09011 24868353 PMC4027696

[35] Bostantjopoulou S, Katsarou Z, Michael M, et al. Reversible parkinsonism due to chronic bilateral subdural hematomas. J Clin Neurosci 2009;16(3):458–460; doi: 10.1016/j.jocn.2008.03.016 19138853

[36] Suman S, Meenakshisundaram S, Woodhouse P. Bilateral chronic subdural haematoma: a reversible cause of parkinsonism. J R Soc Med 2006;99(2):91–92; doi: 10.1177/014107680609900223 16449784 PMC1360497

[37] Kivi A, Trottenberg T, Kupsch A, et al. Levodopa-responsive posttraumatic parkinsonism is not associated with changes of echogenicity of the substantia nigra. Mov Disord 2005;20(2):258–260; doi: 10.1002/mds.20323 15551348

[38] O'Suilleabhain P, Dewey RB Jr. Movement disorders after head injury: diagnosis and management. J Head Trauma Rehabil. 2004;19(4):305–313; doi: 10.1097/00001199-200407000-00005 15263858

[39] Evans AH, Gacinovic S, Costa DC, et al. Parkinsonism due to Kernohan notch: clinical, structural, and functional imaging correlates. Neurology 2004;62(12):2333–2334; doi: 10.1212/01.wnl.0000130350.54120.09 15210916

[40] Matsuda W, Matsumura A, Komatsu Y, et al. Awakenings from persistent vegetative state: report of three cases with parkinsonism and brain stem lesions on MRI. J Neurol Neurosurg Psychiatry 2003;74(11):1571–1573; doi: 10.1136/jnnp.74.11.1571 14617720 PMC1738238

[41] Bhatt M, Desai J, Mankodi A, et al. Posttraumatic akinetic-rigid syndrome resembling Parkinson's disease: a report on three patients. Mov Disord 2000;15(2):313–317; doi: 10.1002/1531-8257(200003)15:2<313::aid-mds1017>3.0.co;2-p 10752583

[42] Wiest RG, Burgunder JM, Krauss JK. Chronic subdural haematomas and Parkinsonian syndromes. Acta Neurochir (Wien) 1999;141(7):753–757; discussion 757–758; doi: 10.1007/s007010050371 10481787

[43] Doder M, Jahanshahi M, Turjanski N, et al. Parkinson's syndrome after closed head injury: a single case report. J Neurol Neurosurg Psychiatry 1999;66(3):380–385; doi: 10.1136/jnnp.66.3.380 10084539 PMC1736270

[44] Sunada I, Inoue T, Tamura K, et al. Parkinsonism due to chronic subdural hematoma. Neurol Med Chir (Tokyo) 1996;36(2):99–101; doi: 10.2176/nmc.36.99 8907012

[45] Weiner WJ, Shulman LM. Post-traumatic movement disorders. Neurology 1995;45(10):1950–1951; doi: 10.1212/wnl.45.10.1950-b 7478011

[46] Krul JM, Wokke JH. Bilateral subdural hematoma presenting as subacute parkinsonism. Clin Neurol Neurosurg 1987;89(2):107–109; doi: 10.1016/0303-8467(87)90184-3 3595016

[47] Nayernouri T. Posttraumatic Parkinsonism. Surg Neurol 1985;24(3):263–264; doi: 10.1016/0090-3019(85)90035-7 4023906

[48] Sandyk R, Kahn I. Parkinsonism due to subdural hematoma. Case report. J Neurosurg 1983;58(2):298–299; doi: 10.3171/jns.1983.58.2.0298 6848695

[49] Sandyk R. Parkinsonism caused by chronic subdural haematoma. A case report. S Afr Med J 1982;61(16):595–596. 7071686

[50] Samiy E. Chronic subdural hematoma presenting a parkinsonian syndrome. J Neurosurg 1963;20:903; doi: 10.3171/jns.1963.20.10.0903 14186088

[51] Grimberg L. Paralysis agitans and trauma. J Nerv Ment Dis 1934;79(1):14–42.

